# The protective effect of CALD identity in the presence of low income on missing teeth of Australian adults over time

**DOI:** 10.1186/s12889-023-17272-5

**Published:** 2024-04-12

**Authors:** Lisa Jamieson, Gloria Mejia, Dandara G Haag, Gustavo H Soares, Liana Luzzi, Xiangqun Ju

**Affiliations:** Australian Research Centre for Population Oral Health, Adelaide Dental School, The University of Adelaide, Adelaide, SA 5005 Australia

**Keywords:** Culturally and linguistically diverse, Social disadvantage, Oral health

## Abstract

**Background:**

‘Culturally And Linguistically Diverse (CALD)’ populations have diverse languages, ethnic backgrounds, societal structures and religions. CALD populations have not experienced the same oral health benefits as non-CALD groups in Australia. However, the socio-demographic profile of Australian CALD populations is changing. This study examined how household income modifies the oral health of CALD and non-CALD adults in Australia.

**Methods:**

Data were from two National Surveys of Adult Oral Health (NSAOH) conducted in 2004-06 (NSAOH 2004-06) and 2017-18 (NSAOH 2017-18). The outcome was self-reported number of missing teeth. CALD status was identified based on English not the primary language spoken at home and country of birth not being Australia. Social disadvantage was defined by total annual household income. Effect-measure modification was used to verify differences on effect sizes per strata of CALD status and household income. The presence of modification was indicated by Relative Excess Risk due to Interactions (RERIs).

**Results:**

A total of 14,123 participants took part in NSAOH 2004-06. The proportion identifying as CALD was 11.7% and 56.7% were in the low-income group, and the mean number of missing teeth was 6.9. A total of 15,731 participants took part in NSAOH 2017-18. The proportion identifying as CALD was 18.5% and 38.0% were in the low-income group, and the mean number of missing teeth was 6.2. In multivariable modelling, the mean ratio (MR) for CALD participants with low household income in 2004-06 was 2% lower than the MR among non-CALD participants with high household income, with the RERI being − 0.23. Non-CALD participants from lower income households had a higher risk of having a higher number of missing teeth than low income CALD individuals (MR = 1.66, 95%CI 1.57–1.74 vs. MR = 1.43 95%CI 1.34–1.52, respectively). In 2017-18, the MR for CALD participants with low household income was 3% lower than the MR among non-CALD participants with high household income, with the RERI being − 0.11. Low income CALD participants had a lower risk of missing teeth compared to their non-CALD counterparts (MR = 1.43, 95% CI 1.34–1.52 vs. MR = 1.57, 95% CI 1.50–1.64).

**Conclusions:**

The negative RERI values indicate that the effect-measure modification operates in a negative direction, that is, there is a protective element to being CALD among low income groups with respect to mean number of missing teeth.

## Introduction

Australia has one of the most diverse populations globally, with data from the 2021 Census indicating that 52% of people living in Australia were either born overseas or had a parent born overseas [1]. The top five countries of birth outside Australia were England, India, China, New Zealand and the Philippines [1]. In 2021, around one in five people living in Australia (22%) spoke a language other than English at home. The phrase ‘culturally and linguistically diverse (CALD)’ is a broad term used to describe communities with diverse languages, ethnic backgrounds, nationalities, traditions, societal structures and religions [2, 3]. It is the preferred term used by Australian service providers and agencies, with a widely used definition referring to those born overseas and speaking languages other than English at home [2]. Historically, CALD populations have not experienced the same health benefits as non-CALD groups in Australia. Many face challenges when navigating the health and welfare system, with language barriers, lower health literacy, and unfamiliar structures putting CALD groups at greater risk of poorer quality health care, service delivery and poorer health outcomes compared with other Australians.

However, in Australia, there are many protective benefits in being CALD, especially among older CALD participants. Many CALD individuals have a strong sense of identity; large families; a deep understanding of their own culture, language, history and traditions; and a robust social and community network. There is often a culture of ‘giving back’ which creates increased empathy and understanding of others. This may buffer negative impacts of stress and social isolation, promote optimal mental health [4], and contribute to strong social and emotional wellbeing. Some CALD groups, for example, Mediterranean and Asian populations, have healthier dietary practices, leading to lower rates of obesity, diabetes and heart disease [5, 6].

In recent decades in Australia, through Government initiatives including the Global Talent visa program [7], an increasing proportion of newly arrived CALD populations are highly educated, with an internationally recognised record of professional and academic achievement. This frequently translates into success in completing high-level tertiary education attainment, and in obtaining high paying and permanent employment. Many have benefitted from social advantage and optimal health in their country of origin, which is able to be continued upon migration to Australia. The social, economic and health profile of CALD populations is thus changing.

Oral health is socially patterned, with the highest burden of unmet dental need being experienced by society’s most disadvantaged groups. Because of inequities in both access to, and cost of, dental service provision for socially disadvantaged groups, diseased teeth are often extracted even when restorative options are available. Missing teeth thus becomes a marker of inequitable dental service provision that has been, regrettably, higher among CALD groups at a population level [8]. Given the increasing heterogeneity of CALD groups in Australia, and the strong role of economic advantage in oral health outcomes, it is pertinent to examine the influence of CALD and social disadvantage on oral health through stratified analysis to provide evidence that is more informed and policy relevant. This study therefore aimed to investigate how household income modifies the oral health of CALD and non-CALD adults over time in Australia, using population-level data. The study hypothesis was that CALD status would be protective across time in the presence of low income with respect to mean number of missing teeth.

## Methods

### Study design and sample selection

Data were from two National Surveys of Adult Oral Health (NSAOH) conducted in 2004-06 (NSAOH 2004-06) and 2017-18 (NSAOH 2017-18) (Chrisopoulos et al., 2019; Slade et al., 2007) [9, 10]. In each survey, representative samples of adults were drawn through a three-stage, stratified sample design within metropolitan and regional areas in each state/territory. The first stage selected a sample of postcodes from all in-scope postcodes in Australia. The second stage selected households within sampled postcodes, with adults aged 15 years and over being randomly selected from each sample household to participate in the final stage. Data were weighted following standard procedures for clustered samples. Both NSAOH 2004-06 and NSAOH 2017-18 were reviewed and approved by the University of Adelaide Human Research Ethics Committee.

### Outcome variables

The outcome variable was self-reported number of missing teeth. This was collected using a computer-assisted telephone interview (CATI) in 2004-06, and CATI or online questionnaire in 2017–18. The specific question asked was: ‘There are 16 teeth, including wisdom teeth in the upper jaw. How many teeth do you have remaining in your upper jaw?’ and ‘There are also 16 teeth, including wisdom teeth in the lower jaw. How many teeth do you have remaining in your lower jaw? Missing teeth was then calculated by subtracting the total number of teeth reported by participants from 32.

### Exposure variable

CALD status was identified based on English not the primary language spoken at home and country of birth not being Australia. Participants were thus categorised as being either: (1) born overseas and not speaking English as the primary language at home or: (2) all others (born overseas and English primary language at home, born in Australia and English not primary language at home, born in Australia and English primary language at home).

### Effect modifier

Household income was the effect modifier and was defined by total annual household income. Categories included low (< AUS$60,000) and high (AUS$60,000+).

### Covariates

Covariates included age, sex, residential location and last dental visit. Age was grouped into ’15–34 years’, ’35 to 54 years’ and ‘55 + years’. Sex was classified as ‘Male’ or ‘Female’. Residential location was categorised into ‘Major city’ or ‘Regional/Remote’. Last dental visit was derived from the question ‘How long ago did you last see a dental professional about your teeth, dentures or gums?’, with responses dichotomized into ‘Less than one year’ and ‘One year or more’.

### Data analysis

Basic descriptive analyses were conducted to ascertain frequencies of variables of interest. Bivariate and multivariable analyses were then conducted to identify effects between the exposure variable (CALD status), effect modifier (household income) and outcome (mean number of missing teeth), accounting for other covariates. Effect Measure Modification (EMM) was used to test if the effect between CALD status (e) and mean number of missing teeth (y) was stronger among low household income groups (q). EMM is present when the association between the exposure and the outcome differs across levels of a second exposure (effect modifier). Following the principles of EMM analysis [11], four categories were created representing all possible combinations between the CALD status indicator and household income. Mean ratios (MR) using generalised linear regressions for mean number of missing teeth for each EMM combination were estimated taking the jointly unexposed (1) as the reference category.

1. No CALD and high household income (MRe0q0); (jointly unexposed)
2. CALD and high household income (MRe1q0);
3. No CALD and low household income (MRe0q1) and;
4. CALD and low household income (MRe1q1) (jointly exposed).

Models included as covariates age, sex, residential location and time since last dental visit. The Relative Excess Risk due to Interactions (RERIs) were then estimated [12]. RERIs are used to assess additive interactions - the effect of one cause (household income) is dependent on the presence of another (CALD status) on an outcome of interest (missing teeth) [13]. RERIs indicate the risk that is in excess of what would be expected if the combination of CALD status and low household income was entirely additive:

$$RERI=M{R}_{{e}_{1}{q}_{1}}-M{R}_{{e}_{0}{q}_{1}}-M{R}_{{e}_{1}{q}_{0}}+M{R}_{{e}_{0}{q}_{0}}$$

A RERI higher than 0 suggests that the effects of the two exposures operating together is higher than that of each added together; a super-additive effect (the effect measure modification is positive). In our analysis, it indicates that the effect of CALD status interacting with low household income is higher than the sum of the independent effects of CALD status and low household income. A RERI of 0 suggests no effect-measure modification is present, whilst a negative value suggest the effect-measure modification operates in a negative direction [14]. RERIs are interpreted by the direction in which the effect-measure modification occurs, as opposed to RERI size per se [11].

SAS version 9.4 was used for all analyses. Missing data was imputed under the assumption that data was missing at random using the Fully Conditional Specification method with linear regression for continuous variables. All missing data were imputed.

## Results

A total of 14,123 participants took part 2004-06 (Table 1). The proportion identifying as CALD was 11.7% and 56.7% were in the low-income group. Just over one-third (36%) were aged 35 to 54 years (Table 2). The proportion of males was 49% and 33% resided in regional or remote locations. Around 41% had last visited a dentist 12 or more months previously. The mean number of missing teeth was 6.9. The proportion of participants who were not CALD and high income was 40%. Within this group, a higher proportion were aged 35 to 54 years, were male, resided in a major city, last visited a dentist less than 12 months previously. The mean number of missing teeth was 3.9. Around 4% of participants were CALD and high income. Within this group, a higher proportion were aged 35 to 54 years and resided in a major city. The mean number of missing teeth was 3.5. The proportion of participants who were not CALD and low income was 50%. Within this group, a higher proportion were aged 55 + years, were female, resided in regional and remote locations and last visited a dentist over 12 months ago. The mean number of missing teeth was 9.8. The proportion of participants who were CALD and low income was 7%. Within this group, a higher proportion were aged 55 + years and resided in a major city. The mean number of missing teeth was 7.4.

**Table 1:** Sample characteristics by CALD status and household income (weighted)

	NSAOH 2004-06 (N = 14,123)	NSAOH 2017-18 (N = 15,731)
**CALD status**		
CALD	11.7 (10.9–12.5)	18.5 (17.5–19.6)
Non-CALD	88.3 (87.5–89.1)	81.5 (80.4–82.5)
**Household income**		
Low (<$60,000)	56.7 (55.6–57.9)	38.0 (36.8–39.1)
High (≥$60,000)	43.2 (42.1–44.4)	62.0 (60.9–63.2)

**Table 2:** Associations between CALD^a=^ and low income characteristics among Australian adults across time, NSAOH 2004-06 and NSAOH 2017-18, weighted estimates

	NSAOH 2004-06 (N = 14,123)					NSAOH 2017-18 (N = 15,731)				
	Total sample	No CALD, no low income	CALD, no low income	No CALD, low income	CALD, low income	Total sample	No CALD, no low income	CALD, no low income	No CALD, low income	CALD, low income
	% (95% CI)	% (95% CI)	% (95% CI)	% (95% CI)	% (95% CI)	% (95% CI)	% (95% CI)	% (95% CI)	% (95% CI)	% (95% CI)
**Total**	100.0	39.5 (38.2–40.7)	4.1 (3.5–4.6)	49.7 (48.4–50.9)	6.8 (6.2–7.4)	100.0	52.2 (50.8–53.6)	10.1 (9.1–11.0)	30.9 (29.7–32.1)	6.8 (6.0-7.7)
**Age group (years)**										
15 to 34	34.8 (33.7–35.9)	48.5 (46.0-51.1)	4.9 (3.7–6.1)	40.1 (37.6–42.6)	6.4 (5.2–7.7)	34.5 (33.5–35.6)	62.2 (59.7–64.7)	10.2 (8.6–11.9)	23.7 (21.5–25.8)	3.9 (2.8-5.0)
35 to 54	35.5 (34.5–36.5)	49.1 (47.2–50.9)	5.1 (4.2–5.9)	39.9 (38.1–41.7)	6.0 (5.1–6.9)	32.6 (31.5–33.6)	60.9 (58.5–63.3)	15.3 (13.4–17.2)	17.9 (16.1–19.7)	5.9 (4.4–7.4)
55 + years	29.7 (28.8–30.6)	16.8 (15.3–18.4)	1.9 (1.3–2.5)	73.0 (71.3–74.2)	8.3 (7.2–9.3)	32.9 (31.9–33.8)	32.2 (30.4–34.0)	4.3 (3.2–5.4)	52.6 (50.5–54.6)	10.9 (9.2–12.6)
**Sex**										
Male	49.4 (48.3–50.4)	43.1 (41.1–45.0)	4.5 (3.6–5.4)	46.1 (44.2–48.1)	6.3 (5.4–7.3)	49.2 (48.1–50.3)	55.2 (53.1–57.2)	11.5 (10.1–12.9)	26.5 (24.8–28.2)	6.8 (5.7-8.0)
Female	50.6 (49.6–51.7)	35.9 (34.3–37.4)	3.6 (3.0-4.3)	53.2 (51.6–54.7)	7.3 (6.5–8.1)	50.8 (49.7–51.9)	49.2 (47.4–51.0)	8.6 (7.3–9.9)	35.4 (33.7–37.1)	6.8 (5.6-8.0)
**Residential location**										
Regional/remote	33.1 (32.1–34.1)	33.2 (31.2–35.2)	0.9 (0.5–1.2)	64.1 (62.1–66.1)	1.9 (1.4–2.4)	28.2 (27.3–29.1)	54.2 (52.1–56.3)	2.1 (1.6–2.6)	41.9 (39.8–44.0)	1.8 (1.2–2.4)
Major city	66.9 (65.9–67.9)	42.9 (41.3–44.5)	5.8 (5.0-6.6)	41.7 (40.2–43.2)	9.6 (8.6–10.5)	71.8 (70.9–72.7)	51.2 (49.5–53.0)	13.9 (12.6–15.2)	25.6 (24.2–27.1)	9.2 (8.0-10.4)
**Last dental visit**										
12 + months ago	40.6 (39.6–41.7)	34.6 (32.6–36.5)	3.2 (2.5-4.0)	55.4 (53.4–57.3)	6.8 (5.8–7.8)	43.6 (42.5–44.7)	47.6 (45.6–49.6)	10.1 (8.6–11.5)	34.0 (32.2–35.9)	8.3 (6.9–9.7)
<12 months ago	59.4 (58.3–60.4)	43.0 (41.3–44.6)	4.7 (3.9–5.4)	45.5 (44.0-47.1)	6.8 (6.0-7.6)	56.4 (55.3–57.5)	56.0 (54.2–57.9)	10.1 (8.8–11.3)	28.3 (26.7–29.9)	5.6 (4.6–6.6)
	**Mean (95% CI)**	**Mean (95% CI)**	**Mean (95% CI)**	**Mean (95% CI)**	**Mean (95% CI)**	**Mean (95% CI)**	**Mean (95% CI)**	**Mean (95% CI)**	**Mean (95% CI)**	**Mean (95% CI)**
**Mean number missing teeth**	6.9 (6.8–7.1)	3.9 (3.7-4.0)	3.5 (2.9–4.1)	9.8 (9.5–10.1)	7.4 (6.6–8.1)	6.2 (6.1–6.3)	4.3 (4.2–4.5)	3.6 (3.3-4.0)	9.7 (9.4–10.0)	8.8 (7.9–9.8)

A total of 15,731 participants took part 2017-18 (Table 1). The proportion identifying as CALD was 18.5% and 38.0% were in the low-income group. Just over one-third (35%) were aged 15 to 34 years (Table 2). The proportion of males was 49% and 28% resided in regional or remote locations. Around 44% had last visited a dentist 12 or more months previously. The mean number of missing teeth was 6.2. The proportion of participants who were not CALD and high income was 52%. Within this group, a higher proportion were aged 15 to 54 years, were male and last visited a dentist less than 12 months previously. The mean number of missing teeth was 4.3. Around 10% of participants were CALD and high income. Within this group, a higher proportion were aged 35 to 54 years, were male and resided in a major city. The mean number of missing teeth was 3.6. The proportion of participants who were not CALD and low income was 31%. Within this group, a higher proportion were aged 55 + years, were female, resided in regional and remote locations, and last visited a dentist over 12 months ago. The mean number of missing teeth was 9.7. The proportion of participants who were CALD and low income was 7%. Within this group, a higher proportion were aged 55 + years, resided in a major city and last visited a dentist 12 or more months previously. The mean number of missing teeth was 8.8.

In multivariable modelling, after adjusting for covariates, the mean ratio for participants who identified as CALD and with low household income in 2004-06 was 2% lower than the ratio among those who did not identify as CALD and with high household income, with the RERI being − 0.23 (Table 3). The mean ratio for participants who identified as CALD and with low household income in 2017-18 was 3% lower than the ratio among those who did not identify as CALD and with high household income, with the RERI being − 0.11. While both CALD and non-CALD individuals from low income households were at substantially higher risk of having a higher number of missing teeth, our findings show that the effect of low income on mean number of missing teeth was less pronounced among the CALD community compared to their non-CALD counterparts across the 2004-06 (MR = 1.43 95% CI 1.34–1.52 vs. MR = 1.66, 95%CI 1.57–1.74, respectively) and the 2017-18 datasets (MR = 1.43, 95% CI 1.34–1.52 vs. MR = 1.57, 95% CI 1.50–1.64).

**Table 3:** Effect measure modification of income on the effect between CALD and mean number of missing teeth among Australian adults between 2004-06 and 2017-18

**NSAOH 2004-06**				
**No CALD**			**CALD**	
Household income	Mean MT (95% CI)	Mean Ratio (95% CI)	Mean MT (95% CI)	Mean Ratio (95% CI)
Low (<$60,000)	9.8 (9.5–10.1)	1.66 (1.57–1.74)	7.4 (6.6–8.1)	1.43 (1.33–1.54)
High (< 60,000+)	3.9 (3.7-4.0)	1 (ref)	3.5 (2.9–4.1)	0.98 (0.84–1.16)
Relative Excess Risk due to Interaction: -0.23 (95% CI: -0.36, -0.08) Mean ratios adjusted for: age, sex, residential location and last dental visit				
**NSAOH 2017-18**				
**No CALD**			**CALD**	
Household income	Mean MT (95% CI)	Mean Ratio (95% CI)	Mean MT (95% CI)	Mean Ratio (95% CI)
Low (<$60,000)	9.7 (9.4–10.0)	1.57 (1.50–1.64)	8.8 (7.9–9.8)	1.43 (1.34–1.52)
High (< 60,000+)	4.3 (4.2–4.5)	1 (ref)	3.6 (3.3-4.0)	0.97 (0.88–1.07)
Relative Excess Risk due to Interaction: -0.11 (95% CI: -0.19, -0.04). Mean ratios adjusted for: age, sex, residential location and last dental visit.				

The negative RERI values suggest that the effect-measure modification operates in a negative direction, that is, there is a protective element to being CALD among low income groups with respect to mean number of missing teeth. That is, the joint association of CALD identification and low household income did not surpass the sum of their separate parts with the oral health outcome of interest in each of the time periods observed.

## Discussion

This study tested the hypothesis that CALD identity would be protective across time in the presence of social disadvantage in terms of poor oral health outcomes. Our findings support this hypothesis, with CALD participants in low income groups having fewer missing teeth than their non-CALD counterparts in low income groups across both time points. Indeed, in 2017-18, the mean number of missing teeth for CALD participants in the high-income group (3.6) was less than that reported for high income non-CALD participants (4.3). The findings suggest that there are other social determinants with a much greater influence on poor oral health outcomes than CALD status per se, with the group most disadvantaged being non-CALD and low household income – a higher proportion of whom were older, female, residing in regional/remote locations who had not visited a dentist in over 12 months. At a population level, this group had, on average, 6 more missing teeth across both time points than their high-income counterparts, irrespective of CALD status.

The protective effect of identifying as CALD in the presence of low income is supported by the literature. Previous research [15] has shown that CALD groups are more likely to be in precarious employment in certain sectors, such as aged care or low-income job. Financial barriers were associated with dental care avoidance, with evidence showing that inequities in dental care utilization resulting in more severe oral diseases and receipt of invasive treatments, such as tooth extraction [16, 17]. Higher income earners experience fewer financial barriers to dental care utilization [18], resulting in better oral health and less tooth loss [19]. With increased pathways for CALD populations to navigate health services, particularly in cities, including translation services and transport support, the impacts in terms of poor oral health are likely to improve across time.

What the findings do highlight are the abject disadvantages experienced by older, low income women living in regional/remote locations; a compounding of social oppressions that will lead to increased oral health inequities over time. The availability of affordable and accessible dental health services for this group is sub-optimal across each of Australia’s states and territories, with dental public health waitlisting being up to two years in some jurisdictions [20], and most requiring some form of co-payment. When teeth have reached an advanced stage of disease, often extraction is the only alternative (more complex treatments such as root canal therapy and crown/bridges/implants are not available in the public sector) [20].

Limitations of the study include the primary outcome, mean number of missing teeth, being self-reported. However, we undertook sensitivity analysis to confirm that the estimates of clinically assessed number of missing teeth closely matched those calculated. **In addition, number of missing teeth was based on 32 total teeth, when some adults may not have had wisdom teeth or wisdom teeth that had not yet erupted. However, this bias would be evenly distributed across the four groups, so unlikely to have a marked impact on the findings.** Our definition of CALD status (English not primary language spoken at home, and not born in Australia) was more stringent than what other CALD researchers have used, leading to a small proportion in our samples identifying as CALD. The household income variable used was not equivalised, meaning it did not account for how many people in the household were dependent on that income. The median split cut-point of $61,000 AUD may also not truly reflect social advantage vs. disadvantage.

In summary, using population estimates across two time points, our findings supported a protective benefit of CALD **identity** in the presence of low income in terms of mean number of missing teeth. The groups identified as being most at risk of poor oral health outcomes were older women living in regional locations who had not accessed a dentist in over 12 months.

## Data Availability

The datasets used and analysed in this study are available from the corresponding author on reasonable request.
